# Differential Development of Children’s Understanding of the Cardinality of Small Numbers and Zero

**DOI:** 10.3389/fpsyg.2018.01636

**Published:** 2018-09-25

**Authors:** Silvia Pixner, Verena Dresen, Korbinian Moeller

**Affiliations:** ^1^Institute of Psychology, UMIT – Private University for Health Sciences, Medical Informatics and Technology, Hall in Tirol, Austria; ^2^Leibniz-Institut für Wissensmedien, Tübingen, Germany; ^3^LEAD Graduate School & Research Network and Department of Psychology, University of Tübingen, Tübingen, Germany

**Keywords:** numerical development, cardinality principle, counting, knower level, visuospatial abilities, language abilities

## Abstract

Counting and the understanding of cardinality are important steps in children’s numerical development. Recent studies have indicated that language and visuospatial abilities play an important role in the development of children’s cardinal knowledge of small numbers. However, predictors for the knowledge about *zero* were usually not considered in these studies. Therefore, the present study investigated whether the acquisition of cardinality knowledge on small numbers and the concept of *zero* share cross-domain and domain-specific numerical predictors. Particular interest was paid to the question whether visuospatial abilities – in addition to language abilities – were associated with children’s understanding of small numbers and *zero*. Accordingly, we assessed kindergarteners aged 4 to 5 years in terms of their understanding of small numbers and *zero* as well as their visuospatial, general language, counting, Arabic number identification abilities, and their finger number knowledge. We observed significant zero-order correlations of vocabulary, number identification, finger knowledge, and counting abilities with children’s knowledge about *zero* as well as understanding of the cardinality of small numbers. Subsequent regression analyses substantiated the influences of counting abilities on knowledge about *zero* and the influences of both counting abilities and finger knowledge on children’s understanding of the cardinality of small numbers. No significant influences of cross-domain predictors were observed. In sum, these results indicate that domain-specific numerical precursor skills seem to be more important for children’s development of an understanding of the cardinality of small numbers as well as of the concept of *zero* than the more proximal cross-domain abilities such as language and visuospatial abilities.

## Introduction

Counting is an important step in children’s numerical development (cf. Fuson, 1988). At an age between 2 and 3 years, children usually start learning the sequence of number words (one, two, three, etc.). In the beginning, children often confuse the sequence of number words but soon learn to recite the number words in the appropriate order. In the present study, we were interested in the development of early numerical abilities in children with a specific focus on their understanding of cardinality, i.e., their understanding of the fact that each number word represents a specific quantity. In particular, we aimed at identifying relevant predictors of children’s early understanding of the cardinality of small numbers. In the context of small numbers, it is interesting to note that children seem to acquire the concept of *zero* in a way that is different from how they acquire the concept of other small numbers. For instance, children hardly ever start counting at *zero*. Thus, the development of children’s understanding of the cardinality of small numbers and the concept of *zero* seem to differ. However, while children’s understanding of the cardinality of small numbers has been investigated quite well, this is not the case for their acquisition of the concept of *zero*. Therefore, we paid specific attention to the development of children’s knowledge of *zero*. In particular, we were interested in whether the same variables that predict children’s mastery of the cardinality of small numbers larger than *zero* (i.e., numbers 1–7) also predict the acquisition of the concept of *zero*.

In the following, we first give a brief introduction on the development of children’s understanding of the cardinality of small natural numbers and the specificity of the concept of *zero* before going into the details of the current study.

### Development of Children’s Understanding of Cardinality

Children’s first attempts at counting often turn out to be a numerically meaningless recitation of number words (Fuson, 1988). At this stage, children may not yet understand that the number word *two* refers to the numerosity of a set of two objects. In addition to keeping to the correct order of number words, it is also necessary to follow the principle of one-to-one correspondence for successful counting (Gelman and Gallistel, 1978). Finally, to understand that the number word named last actually represents the number of items in the set counted, children need to understand the concept of cardinality.

Most children between 2 and 3 years of age still have trouble in fully understanding cardinality (Fuson, 1988). Interestingly, recent studies have indicated that children do not acquire an understanding of cardinality for all numbers at the same time. Rather, this seems to be a step-by-step process. In the first step, children acquire the cardinal meaning of *one* while all other numbers are simply considered larger than *one* (e.g., Sarnecka and Carey, 2008). When a child at this so-called *one*-knower level (i.e., she/he only understands the cardinal meaning of *one*) is asked to give an experimenter two or three objects, the child will most probably pass more than one object without further differentiating between these larger numerosities. Some months later, children reach the *two*-knower level (i.e., she/he understands the cardinal meaning of *one* and *two*; Sarnecka and Carey, 2008). This level is followed by the *three*-knower level and then by the *four*-knower level, the understanding of each new number being assumed to build on children’s understanding of the previous numbers (e.g., Sarnecka et al., 2007). Children at these levels (*one* to *four*) have been termed as “subset-knowers” (Le Corre and Carey, 2007).

After the *five*-knower level has been reached, most children show a change in their further development of understanding the cardinal meaning of number words. Suddenly, they seem to be able to generate the right cardinality for *five* and larger numbers. At this level, children are identified as “cardinality-knowers” (Sarnecka and Carey, 2008). Sarnecka and Carey (2008) explain that cardinality-knowers differ qualitatively from subset-knowers because cardinality-knowers *understand* how counting works. At the age of around three-and-a-half years, children usually master the significance of cardinality by realizing that a set of five objects, labeled with the number word *five*, can also be counted *one, two, three, four, and five* (Mix, 2009).

Importantly, there is accumulating evidence that the above described development of children’s understanding of the cardinality of small numbers is influenced by both cross-domain as well as domain-specific numerical abilities (e.g., LeFevre et al., 2010). In particular, the influences of language (e.g., Carey, 2004; Negen and Sarnecka, 2012) as well as visuospatial abilities (e.g., Newcombe et al., 2015 for a review) were observed. Therefore, we specifically considered these two cross-domain abilities when investigating the predictors of children’s understanding of the cardinality of small numbers and *zero*. In the following, we will first summarize the evidence of the influence of language and visuospatial abilities on children’s understanding of the cardinality of small numbers and *zero* before considering the influences of domain-specific numerical predictors.

### Cross-Domain Factors Influencing the Understanding of Cardinality: Language

In this context, the question arises whether there are meaningful predictors of children’s understanding of cardinality as sketched above based on knower levels. Recent studies have indicated that language abilities may play an important role. Negen and Sarnecka (2012) found that understanding the cardinal meaning of the first number words was associated with the development of children’s vocabulary: the larger a child’s vocabulary, the better her/his cardinal number knowledge. Interestingly, this association may be influenced by the fact that linguistic markers might well corroborate differentiating between *one* and *more*. For example, in the English language, any number larger than *one* is usually followed by a plural noun, with “-s” added to the word representing the counted objects (e.g., *one* car but *two* or *more* car*s*). At such an early stage, these markers may help children differentiate between *one* (singular) and *more* (plural).

This hypothesis is backed by the findings of Sarnecka et al. (2007) who observed that children who speak Japanese, which is a so-called classifier language with no such singular–plural distinction, take longer to understand the cardinality of *one* than English- or Russian-speaking children whose languages differentiate explicitly between singular and plural. Importantly, additional analyses by Sarnecka et al. (2007) indicated that parents from all three countries used number words in a comparable manner when interacting with their children. Moreover, Barner et al. (2007) found that English-speaking children distinguished the quantity of *one* and *more* at about 22–24 months of age. Interestingly, this corresponded to the same age that parents reported that their children began using plural nouns. Another interesting point is that some languages differ in marking the plural of small magnitudes (2–4) and all magnitudes above *five*. For example, in Slovakian people say: one jablko (apple), two jablka (apples), and five jablk (apples, but with another ending). Thus, it might be assumed that in these languages additional hints are given by these changing plural markers for the important development of children’s understanding of the cardinality of small numbers.

This evidence corroborates the claim that language plays a crucial role in children’s acquisition of cardinality knowledge of small numbers (Barner et al., 2009). In particular, language was argued to provide essential mental “glue” that enables the human mind to assemble new complex concepts from simple primitives (Spelke, 2003; Condry and Spelke, 2008). This raises the obvious question whether the continuum of numbers is derived from language or whether there are other factors influencing children’s early numerical development.

### Further Cross-Domain Factors Influencing the Understanding of Cardinality: Visuospatial Abilities

Apart from language abilities, there is also compelling evidence suggesting that visuospatial skills may be associated with children’s numerical development (e.g., Ansari et al., 2003; Gunderson et al., 2012; LeFevre et al., 2013; Patro et al., 2014; Pixner et al., 2017 for a review on spatial-numerical association in preliterate children; see Newcombe et al., 2015 for a review on the intertwined development of spatial and numerical skills). This seems reasonable as Newcombe et al. (2015) argue that space and number have a mutual basis, i.e., the generalized magnitude system that is resorted to both simple spatial and numerical tasks. Furthermore, it is supposed that there is a spatial representation of number magnitudes often referred to by the metaphor of a *mental number line*. On the mental number line, numbers are assumed to be represented in ascending magnitude order from left to right (at least in Western countries; e.g., Dehaene et al., 1993). As such, the mental number line represents a combination and integration of spatial and numerical concepts.

Nevertheless, there are only very few studies that pay specific attention to the association between children’s visuospatial abilities and their development of cardinal number knowledge during early childhood (e.g., Ansari et al., 2003). Most of the related research examined primary school children and mainly considered the development of the mental number line. Yet, tasks usually employed to assess the mental number line often require both the cardinal knowledge of number magnitudes as well as visuospatial abilities (e.g., the number line estimation task in which a target number has to be located on a number line of which only the start and end points are given, e.g., Siegler and Opfer, 2003).

In this context, Gunderson et al. (2012) observed that in first and second graders, spatial skills predicted the improvement in number line estimation over the course of the school year. In addition, children’s spatial skills also predicted later approximate calculation abilities. These findings are substantiated by the results of training studies in which spatial-numerical trainings were more effective than non-spatial control training in enhancing kindergartners’ number line estimation as well as counting performance (Fischer et al., 2011, 2015; Dackermann et al., 2016 for overviews of spatial-numerical trainings). In line with this, Siegler and Ramani (2008) found that knowledge of numerical quantities in 4-year-olds improved significantly when they played board games that involved a physical realization of the mental number line (i.e., moving a token as many steps to the right as there were points on a dice).

Taken together, this suggests that cross-domain abilities such as (visuo-)spatial abilities as well as language abilities (see above) seem to play a crucial role in children’s numerical development. Of course, children’s numerical development is also influenced considerably by domain-specific numerical predictors as described in the following.

### Domain-Specific Basic Numerical Factors Influencing the Understanding of Cardinality

In addition to cross-domain abilities such as language and visuospatial abilities, it was observed that numerical competencies such as children’s understanding of cardinality are also influenced by other domain-specific basic numerical competencies such as counting (e.g., Aunola et al., 2004), the ability to identify and name number symbols (e.g., Schmidt, 1982), as well as finger-based numerical representations (e.g., Noel, 2009). While the association is obvious for counting and number identification, the influence of finger-based representations needs a brief introduction. For instance, finger-based number gestures (e.g., thumb, index, and middle finger stretched out to represent three) serve as an important bridge between preverbal mental representation of numbers and number words (e.g., Gunderson et al., 2015; Roesch and Moeller, 2015 for a theoretical discussion). Usually, at the age of two, children begin to use such gestures while counting (Gelman and Gallistel, 1978), exactly at the same time as they begin to understand the cardinality of numbers. This led us to consider finger-based numerical representations when investigating the development of children’s understanding of cardinality.

As already mentioned above, a specific focus of the current study was on examining children’s understanding of the concept of *zero* by evaluating possible predictors for the acquisition of the concept of *zero*. In particular, we aimed at evaluating whether cross-domain language and/or visuospatial abilities as well as domain-specific numerical factors also play an important role in the acquisition of the concept of *zero*. Or is the mastery of cardinality of small numbers necessary to understand the concept of *zero*?

### The Specific Role of *Zero*

From an evolutionary point of view, *zero* is a rather “young” number (Butterworth, 1999). The use of *zero* was reported first in about 300 BC (Seife, 2000), even though people had used numbers in everyday life long before. To date, only few studies have examined the processing of *zero* and its development in detail (see Nieder, 2016 for a recent review on the emergence and the development of *zero*). There is evidence that processing *zero* is unique in both children as well as adults (Wellman and Miller, 1986; Brysbaert, 1995). Yet, difficulties in understanding *zero* may not only refer to the numerical value of *zero*, but may originate from difficulties at a more general level of understanding the concept of *nothing*. Wynn and Chiang (1998) analyzed the development of the concept of *no object* in a series of experiments with infants. In these experiments, a single item ‘magically’ appeared/disappeared in a location in which no/an item had been shown before. Infants were not surprised when an object magically appeared. However, they were irritated by the magical disappearance of an object from its former location. From these findings, Wynn and Chiang (1998) concluded that 8-months-old infants were unable to understand *no objects*.

Moreover, Wellman and Miller (1986) reported that children first learn to identify the symbol of *zero* without actually understanding what this symbol means semantically. Only later on, children are assumed to learn that *zero* represents *nothing*, but initially without considering it as a numerical value. Therefore, children at this stage may still not understand whether *zero* is more or less than *one*. At the age of 5 to 6 years, at the end of preschool, however, most children understand that *zero* is a numerical concept and do correctly identify it as the smallest natural number (Wellman and Miller, 1986).

When looking at the development of the differentiation between *one*, *two*, and so on as described above, it becomes clear that *zero* is unique. Interestingly, from a linguistic point of view, *zero* is associated with using the plural form of the respective noun in many languages (e.g., *zero* car*s* in English, null Autos in German, etc.), even though *zero* is even less than *one* and is found to the left of *one* on the mental number line. Moreover, *zero* is usually not part of children’s common counting sequence. Mostly, children start counting at *one* and not at *zero*. Moreover, unlike other integers, *zero* does not represent the presence of a quantity, but its absence. Accordingly, these specificities may influence children’s understanding of the cardinal meaning of *zero*. For instance, in a magnitude comparison, four-year-old children were just as likely to indicate that *zero* is larger than *three* as vice versa (Merritt and Brannon, 2013). This was examined in a non-symbolic numerosity comparison task, in which trials with *no* objects were presented. Children had to decide, on which one of two pictures they could see more objects. From this result, Merritt and Brannon (2013) concluded that *zero* is represented on the same numerical continuum as other natural numbers at the age of about 4 years.

However, not only for children but also for adults the representation of *zero* seems to be different from the representation of other small numbers. For instance, Brysbaert (1995) found that reading times for small integers (e.g., one, two, or three) were significantly shorter than the reading time for *zero*. This indicates that processing of *zero* differs substantially from processing of other integers and might be based on other principles (Brysbaert, 1995). Grounded on this and other findings, Pinhas and Tzelgov (2012) concluded that *one* may be considered the innately smallest number (Leslie et al., 2008), whereas *zero* represents a later and the smallest culturally acquired number.

Another role of *zero* is its placeholder function in multidigit numbers. Many studies have documented that children, and adults too, have difficulties in understanding the placeholder function of *zero* (Brown, 1981; Crooks and Flockton, 2002). Further problems representing *zero* were found by Wheeler and Feghali (1983) who observed that adults had more problems completing arithmetic problems when at least one *zero* was involved. Wellman and Miller (1986) inferred that these problems originate from the fact that computations with *zero* usually require the correct application of specific rules (X times 0 is 0, but X plus 0 is X) and thus differ from computations involving other natural numbers.

Considering this representational specificity of *zero,* one cannot be sure that language that was supposed (and observed) to predict children’s acquisition of the cardinality of small numbers also predicts children’s understanding of the concept of *zero*. As mentioned above, nouns linked with *zero* are linguistically marked as plural (e.g., *zero* cars) in many languages. Accordingly, children might misinterpret *zero* as representing a quantity larger than *one*. Therefore, we suggest that children refer to other sources of information to correctly understand the concept of *zero*. In particular, visuospatial abilities associated with processing of spatial attributes of the mental number line (i.e., *zero* being smaller and thus located to the left of *one* on the mental number line) or basic numerical abilities, such as understanding the cardinality of small numbers, may be recruited in this process.

Taken together, the present study set out to evaluate the possibly differential association of cross-domain abilities such as language and visuospatial skills of children with their understanding of the cardinality of small numbers as observed in previous studies while also considering the influences of domain-specific basic numerical abilities (i.e., counting, number identification, and finger-based representations). We hypothesized that the influences of domain-specific basic numerical competencies should outweigh those of cross-domain abilities because they allow for a more specific prediction of later numerical skills. However, going beyond previous studies, we were specifically interested in children’s knowledge of *zero* and whether the acquisition of the concept of *zero* is influenced by language, visuospatial, and basic numerical abilities in a way comparable to the cardinality of small natural numbers. As there is only very little research on the development of children’s knowledge of *zero*, it is hard to derive a specific hypothesis. Nevertheless, similar to the case of children’s understanding of the cardinality of small numbers, we would hypothesize that domain-specific numerical predictors should be more important than cross-domain ones.

## Materials and Methods

### Participants and Sample Description

For our study, children were recruited from local public kindergartens around Innsbruck, Austria. Altogether, 65 children (31 boys and 34 girls) were included in this study. Their age ranged from 4 to 5 years (*M* = 4 years, 4 months, *SD* = 3 months). Most of the children (81.5%) were right-handed. All children attended the kindergarten regularly for at least 1 year, were monolingual native speakers of German, and showed no intellectual or language impairments. Written informed consent was obtained from parents prior to the study and children were asked for verbal assent prior to assessment. The study was approved by the Research Committee for Scientific and Ethical Questions at UMIT and school authorities of the state of Tyrol, Austria.

### Procedure and Tasks

Participating children were tested in German in a single one-on-one session in a quiet room in their kindergarten.

The assessment of children’s *numerical and counting* skills comprised four tasks:

(1) Children were asked up to which number they could count. The number sequence formed by each child was transcribed by the experimenter. The task was stopped when a child was obviously uncomfortable about continuing, began to repeat previously used segments, or was not able to continue her/his sequence any further. The largest number for which the counting sequence was correct was considered as the dependent variable.
(2) To identify children’s knower level for the numbers 1–7, as well as their knowledge of *zero*, we used a variant of the Give-N task. As known from previous research, cardinality generalizes for numbers ≥5 (Sarnecka and Carey, 2008). Nevertheless, we tested up to the number 7 to check whether it would be the same or not.
  Quantities were presented randomly and each quantity was presented only once. This was due to our consideration of using Rasch models to analyze the Give-N task for which the repeated presentation of items is not beneficial (see below for the results of the Rasch analyses; Bühner, 2011). Additionally, this made testing sessions shorter and helped keeping children motivated and attentive.
  Children were first requested to take the respective number of stones (0–7) out of a box. All children who mastered the cardinality of *one* but failed for the cardinality of *two* and more were grouped into knower level 1. Similarly, all children who mastered the cardinality of *one* and *two* were considered to be in knower level 2 and so forth. Knowledge of *zero* was the criterion to group the children in the zero-knower or no-zero-knower groups for the later analysis. Importantly, correct scoring of children’s responses to the *zero* item was not trivial because a correct reaction to this item would be doing simply nothing. Hence, whenever a child did not articulate that she/he did nothing on purpose, experimenters were instructed to ask children whether doing nothing/not responding was their answer to this trial. Thereby, we aimed at substantiating evidence on whether or not children understood the meaning of *zero*.
(3) Furthermore, to assess children’s *number identification* abilities, those had to name a numeral that was presented in Arabic form (i.e., 0–7) on a card. Cards were presented randomly and each of it one time. Correct answers were awarded one point resulting in a maximum score of eight points. Sum scores served as the dependent variable.
(4) To identify children’s finger knowledge, the children were asked to present a different configuration of fingers. Quantities between *zero* and 10 were asked. Each quantity was presented one time and in random order. Any numerically correct finger configuration was accepted as a correct answer irrespective of whether the produced finger pattern showed a canonical or non-canonical pattern with respect to the standard German finger counting routine. Again, correct answers were awarded 1 point with a maximum of 11 points. Sum scores served as the dependent variable.

The order of these numerical tasks was counterbalanced across participants as far as possible to prevent sequence effects.

Additionally, we used the *visual-perception* subtest of the Beery-Buktenica Developmental Test of Visual-Motor Integration (VMI; Beery, 2004) to assess the visuospatial abilities in children. This task focuses on the visual discrimination component that was important to us and not on fine motor skills, which are often assessed in similar studies. In this paper-and-pencil-based test, children had to complete up to 16 geometric forms/patterns representing items with increasing complexity. For each item, children had to decide which out of four shapes presented in a response box below the actual item fitted the one shape shown as the actual stimulus. Visual discrimination is needed to solve these items. For each correctly solved item, children were awarded one point summing up to a maximum of 16 points in this task with sum scores serving as the dependent variable. We used this task as it focuses on visual discrimination and, thus, seemed more appropriate to us as a measure of visuospatial processing compared to tasks on visuo-motor integration (e.g., Corsi block) often used in other studies (e.g., LeFevre et al., 2010). Please note that comparable tasks focusing on visual discrimination were previously used by, for instance, Zhang et al. (2014) pursuing a similar research question.

Moreover, to assess the general *language* abilities, the standardized active vocabulary test (Aktiver Wortschatztest for 3- to 5-year-old children; AWST-R, Kiese-Himmel, 2005) was administered (following the approach of Negen and Sarnecka, 2012, on measuring language abilities). In this test, children have to name visually presented objects (nouns) and activities (verbs). The test material consists of 75 picture cards (51 nouns and 24 verbs). For each correctly named object or activity of the presented scenarios, children were awarded one point. In this test, a maximum of 75 points could be achieved. Sum scores were used as the dependent variable.

## Results

### Knower Levels

Results of the Give-N task indicated 1 one-knower, 5 two-knowers, 7 three-knowers, 10 four-knowers, and 42 cardinality-knowers (5, 6, and 7; for more details see Sarnecka and Carey, 2008) in our sample. As to the knowledge of *zero,* we found that 33 children already understood the concept of *zero*, whereas 32 did not yet master this concept.

For the first part of the analyses, children were classified into non-zero-knowers and zero-knowers. For the second part of the analyses, children were classified into groups of subset-knowers and cardinality-knowers. All children on the 1- to 4-knower levels were considered subset-knowers, whereas the others were considered cardinality-knowers (for more details see Sarnecka and Carey, 2008).

Subsequent statistical analyses followed a two-step procedure. In the first step, we evaluated the potential differences between non-zero-knowers and zero-knowers as well as subset-knowers and cardinality-knowers with regard to age, language (vocabulary), and visuospatial abilities as well as number identification, finger knowledge, and counting abilities, and the actual knower level if they understood *zero* and accordingly had the knowledge or non-knowledge of *zero* at the actual knower level, using *t*-tests.

In the second step, we conducted regression analyses to evaluate the predictive value of the above mentioned predictors for knowledge of *zero* as well as children’s cardinality knowledge, that is whether and which of these competencies are relevant for children’s acquisition of the concept of *zero* and the cardinality of small numbers. As regards knowledge of *zero*, we ran a logistic regression analysis predicting zero-knowers vs. non-zero-knowers, whereas a multiple linear regression analysis was conducted to predict children’s knower level reflecting their understanding of the cardinality of small numbers (continuously coded for cardinalities from 1 to 4 plus cardinality-knowers).

In both regression analyses, predictors were considered block-wise. In the first block, non-numeric predictors, vocabulary, and visuospatial perception were incorporated in the regression model. In the second block, basic numerical abilities, number identification, finger knowledge, and counting abilities were included in the model. In the last step, the knower level (continuously coded for cardinalities from 1 to 4 plus cardinality-knowers) or knowledge of *zero* (coded categorically 1 or -1 for successful or not successful understanding of *zero*, respectively) was included. A *p* < 0.05 level of significance for the change in *R*^2^ was applied for the inclusion of the predictors in the regression model.

### Mastery of the Concept of *Zero*

The first part of the analyses addressed children’s knowledge of *zero* in the present sample. Interestingly, 14 out of the 42 cardinality-knowers did not show understanding of *zero*, whereas there were 5 out of 23 subset-knowers who already understood the concept of *zero*. This descriptive analysis shows that there might be a double dissociation between understanding the cardinality of small numbers and understanding the concept of *zero* as there are children in our sample who have already acquired one concept but not the other one or vice versa.

These first indications for differences in children’s understanding of the concept of *zero* and the cardinality of small numbers were substantiated by an analysis of the discrimination of respective items; that means, the item measuring the concept of *zero* may allow for differing discrimination compared to the items for small numbers. The hypothesis of equal item discrimination can be tested in the Rasch model (Rasch, 1960) by applying the so-called pseudo-exact or conditional tests (Ponocny, 2001; Draxler and Zessin, 2015), which are particularly suited for small sample sizes. The results of the conditional tests yielded a *p*-value of 0.044 for the item measuring the concept of *zero* and considerably higher *p*-values for the rest of the items, indicating that *zero* seems to be processed differently. These results are in accordance with the descriptive analysis (see above). Furthermore, a Bayesian analysis according to Draxler (2018) substantiated these results. The obtained posterior distributions indicated the item assessing the understanding of the concept of *zero* as the one with deviating discrimination in comparison to the other items.

Second, we evaluated the differences between non-zero-knowers and zero-knowers in terms of age, vocabulary, visuospatial perception, number identification, counting abilities, finger knowledge, and knower level (cf. **Table 1**). We observed that zero-knowers were significantly better than non-knowers of *zero* at vocabulary, counting abilities, number identification, finger knowledge, and knower level, but not on visuospatial perception.

**Table 1:** Statistical details of the comparison of non-knowers and knowers of *zero*.

	Non-zero-knowers *N* = 32	Zero-knowers *N* = 33		
	Mean *(SE)*	Mean *(SE)*	*t*	*p*
Age	52.47 (0.58)	52.15 (0.45)	*t*(63) = 0.43	0.668
Knower level of numbers	4.44 (0.34)	6.30 (0.23)	*t*(63) = 4.56	<0.001
Visuospatial perception (VMI)	10.50 (0.47)	10.21 (0.45)	*t*(63) = 0.44	0.661
Vocabulary (AWST)	33.75 (1.55)	41.36 (1.64)	*t*(63) = 3.37	0.001
Counting abilities	9.87 (0.83)	15.88 (0.96)	*t*(61^∗^) = 4.69	<0.001
Number identification	3.22 (0.49)	5.30 (0.40)	*t*(63) = 3.29	0.002
Finger knowledge	3.81 (0.27)	4.97 (0.24)	*t*(63) = 3.20	0.002


*SE of the mean given in parenthesis. ^∗^Two children refused this task, but results did not change marginally when children were omitted from all analyses.*

Because no age differences between groups were observed and no significant correlation between age and knowledge of *zero* (*r* = -0.05, *p* = 0.668, see **Table 2** for correlations of other variables), age was no longer considered in the regression.

**Table 2:** Correlation of all variables.

Task	1	2	3	4	5	6	7
1. Vocabulary	1						
2. Visuospatial perception	0.13	1					
3. Number identification	0.37^∗∗^	0.11	1				
4. Finger knowledge	0.26^∗^	0.13	0.63^∗∗^	1			
5. Counting ability	0.33^∗∗^	–0.27^∗^	0.45^∗∗^	0.39^∗∗^	1		
6. Knower level	0.41^∗∗^	0.03	0.57^∗∗^	0.65^∗∗^	0.52^∗∗^	1	
7. Knowledge of *zero*	0.39^∗∗^	–0.06	0.39^∗∗^	0.36^∗∗^	0.51^∗∗^	0.50^∗∗^	1


*^∗^p < 0.05; ^∗∗^p < 0.01.*

In the next step, a logistic regression analysis with knowledge regarding *zero* (successful vs. not successful) as the dependent variable was run. In the first block, we included the predictors visuospatial perception and vocabulary. The model showed a significant goodness of fit [*χ*^2^(2) = 11.55, *p* = 0.003] with a Cox and Snell *R*^2^ value of 0.16 and a Nagelkerke’s pseudo *R*^2^ value of 0.22, which corresponds to a strong effect according to Cohen (1992). Only vocabulary turned out to be a significant predictor with better vocabulary predicting better *zero* knowledge (*b* = 0.098, *SE* = 0.32, odds ratio = 1.103, *p* = 0.003).

In the second block, counting abilities, number identification, and finger knowledge were included in the analysis as additional predictors. Again, the model fit the data significantly [*χ*^2^(5) = 24.58, *p* < 0.001, Cox and Snell *R^2^* = 0.32, pseudo *R^2^* = 0.43, which corresponds to a very strong effect according to Cohen (1992)]. Here, only counting abilities (*b* = 0.199, *SE* = 0.084, odds ratio = 1.22, *p* = 0.017) were a significant predictor. Inspection of beta weights indicated that better *zero* knowledge was associated with better counting abilities. Finger knowledge, number identification, visuospatial perception, and vocabulary did not account for a significant part of the variance.

In the third block, children’s knower level was included in the model. The final model again fit the data significantly well [*χ*^2^(6) = 26.44, *p* < 0.001, Cox and Snell *R^2^* = 0.34, pseudo *R^2^* = 0.46, corresponding to a very strong effect according to Cohen (1992)]. Again, only counting abilities (*b* = 0.178, *SE* = 0.085, odds ratio = 1.19, *p* = 0.036) were found to be a significant predictor of *zero* knowledge. Better counting abilities were associated with better *zero* knowledge. Finger knowledge, number identification, visuospatial perception, vocabulary, and knower level were not considered as meaningful for identifying *zero*-knowers in our sample.

### Children’s Understanding of Cardinality

As can be read from **Table 3**, regarding knower levels we found significant differences between cardinality-knowers and subset-knowers for the vocabulary task, the number identification task, the counting ability task, the finger knowledge task, and knowledge of *zero*. Cardinality-knowers showed higher scores as compared to subset-knowers on all of these tasks. No significant differences were found for visuospatial abilities and with regard to age.

**Table 3:** Statistical details of the comparisons between subset-knowers and cardinality-knowers.

	Subset-knowers *N* = 23	C-knowers *N* = 42		
	Mean *(SE)*	Mean *(SE)*	*t*	*p*
Age	53.09 (0.67)	51.88 (0.42)	*t*(63) = 1.60	0.115
Visuospatial perception (VMI)	10.43 (0.55)	10.31 (0.41)	*t*(63) = 0.18	0.855
Vocabulary (AWST)	32.96 (1.70)	40.17 (1.51)	*t*(63) = 3.00	0.004
Counting abilities	9.19 (1.15)	14.93 (0.81)	*t*(61^∗^) = 4.09	<0.001
Number identification	2.52 (0.52)	5.24 (0.37)	*t*(63) = 4.31	<0.001
Finger knowledge	3.17 (0.29)	5.07 (0.18)	*t*(63) = 5.75	<0.001


*SE of the Mean given in parenthesis. ^∗^Two children refused this task, results did not change when these children were omitted from all analyses.*

Because no age differences between the groups were found and the correlation between age and knower level was not significant (*r* = -0.135, *p* = 0.282, see **Table 2** for the correlation matrix of predictors), age was no longer considered in the regression analyses.

In the next step, a multiple linear regression analysis was conducted to predict children’s knower level reflecting their understanding of the cardinality of small numbers (continuously coded for cardinalities from 1 to 4 plus cardinality-knowers). In the first block, we included the predictors visuospatial abilities and vocabulary. Only vocabulary accounted for a significant part of the variance [*R^2^* = 0.17, *adj. R^2^* = 0.15, *F*(1, 63) = 12.57, *p* = 0.001]. Inspection of beta weights indicated that increases in vocabulary (constant = 2.465; *B* = 0.08, *SE* = 0.02, standardized *ß* = 0.408; *p* = 0.001) were associated with a higher knower level.

In the second block, additionally, counting abilities, number identification, and finger knowledge were considered as predictors in the analysis. In the final model [*R^2^* = 0.47, *adj. R^2^* = 0.46, *F*(2, 58) = 26.16, *p* < 0.001], the predictors counting abilities (constant = 1.485; *B* = 0.10, *SE* = 0.03, standardized *ß* = 0.316; *p* = 0.003) and finger knowledge (*B* = 0.60, *SE* = 0.12, standardized *ß* = 0.506; *p* < 0.001) accounted for a significant part of the variance. Inspection of beta weights indicated that better counting abilities and higher finger knowledge were associated with higher knower level. In contrast, number identification, visuospatial perception, and vocabulary did not account for a significant part of the variance. Please also note that vocabulary was no longer a significant predictor of knower level as soon as either counting abilities or finger knowledge was considered in the model.

In the third block, knowledge of *zero* was included in the model as an additional predictor. However, this did not change the predictors considered in the final regression model. This indicated that children’s knowledge of *zero* did not seem to be predictive of their cardinal number knowledge of small numbers.

## Discussion

The present study aimed at investigating possibly differential prediction of cross-domain abilities such as language skills and visuospatial abilities as well as domain-specific abilities such as counting, finger knowledge, and number identification skills of kindergartners’ understanding of the concept of *zero* and the cardinality of small numbers. In the following, we will elaborate on these points in turn.

As we were particularly interested in the development of the concept of *zero*, our first objective was to identify predictors for children’s understanding of the concept of *zero*. The results of the regression analyses as well as the Rasch analysis showed a significant difference between the understanding of cardinality of small numbers and the concept of *zero*. Descriptive analyses also showed that 14 out of the 42 cardinal-principle-knowers did not show understanding of *zero* whereas 5 out of 23 subset-knowers already understood the concept of zero. This provides further evidence for the claim that cardinality-knowledge for small numbers and zero seems to develop differently. Therefore, we assumed that different processes might be responsible for the development of these two concepts.

Therefore, we first evaluated whether there were group differences on cross-domain (i.e., language and visuospatial abilities) as well as domain-specific numerical variables (i.e., counting skills, number identification, finger knowledge, and knower level) between children who already mastered the concept of *zero* and those (children) who did not. Results indicated the expected significant differences between the two groups in language, counting abilities, number identification, finger knowledge, and children’s knower level. Children who already mastered the concept of *zero* showed better performance on all of the respective abilities, but not with regard to visuospatial abilities.

To evaluate the predictive value of cross-domain (i.e., language and visuospatial abilities) and domain-specific (i.e., counting, number identification, and finger knowledge) variables for children’s understanding of the concept of *zero,* we followed a three-stage procedure with logistic regression analyses. We first incorporated cross-domain variables and observed that language, but not visuospatial abilities, was a relevant predictor for children’s understanding of the concept of *zero*. When considering counting, number identification, and finger knowledge in the second step, only counting skills remained as a significant predictor of children’s understanding of *zero*. Finally, in the third step, the significant influence of counting abilities was prevailing when considering children’s knower level.

These results were only partially in line with our expectations. On the one hand, we found that in the final model, language did not account for a unique part of variance in children’s understanding of the concept of *zero*. In German (i.e., the first language of the children examined in the current study) as well as in English and many other languages, *zero* as a number is followed by the plural form of a noun (e.g., *zero* car*s*). While the plural form correctly indicates the differentiation between *one* and *more* and may thus help children acquire the cardinality principle of small numbers, it may be a hurdle for children’s acquisition of the concept of *zero*. In line with this notion, we did observe that language was no longer a significant predictor of children’s understanding of *zero* as soon as domain-specific numerical variables were considered in the model. In particular, only counting skills were found to be of a significant predictive value for children’s understanding of the concept of *zero*. However, language, but not visuospatial abilities, was a significant predictor when only cross-domain variables were considered. This finding is hard to reconcile with the notion that because of the inconsistencies regarding its language coding, *zero* might rather be internalized by visuospatial representations. In sum, our findings suggest that language in general and language-based specific numerical skills such as counting seem to be significant predictors of children’s early understanding of the concept of *zero*. Thus, these findings indicate that the understanding of the cardinality of small numbers and the concept of *zero* seem to be rather independent of each other.

Apart from investigating the predictors of children’s early understanding of the concept of *zero*, we were interested in children’s understanding of the cardinality in the number range from *one* to *seven*. This was motivated by the findings of Sarnecka and Carey (2008) who claimed significant differences between subset-knowers and cardinality-knowers; that means, children who only internalized the cardinality for a subset of numbers (e.g., 1-, 2-, or 3-knowers) and children who already understood the cardinality of numbers up to *five* and beyond. Our analyses substantiated the expected differences between the two groups in language (vocabulary), counting abilities, number identification, and finger knowledge, but again not with regard to visuospatial abilities.

Comparable to the case of children’s understanding of the concept of *zero*, we then ran regression analyses to evaluate the predictive value of cross-domain (i.e., language and visuospatial abilities) and domain-specific (i.e., counting, number identification, finger knowledge) variables as well as the influence of children’s understanding of the concept of *zero*. In the first step, we considered only cross-domain variables in the regression analysis. We observed that language, but not visuospatial skills, was a relevant predictor for children’s cardinality knowledge. In the next step, we further included domain-specific numerical predictors and found that this led to the observation that language was no longer a significant predictor of children’s understanding of the cardinality of small numbers. Instead, the latter was predicted significantly by children’s counting abilities as well as their finger number knowledge. This is in line with the findings of Reeve and Humberstone (2011) who found a positive association between children’s use of finger-based numerical representations and their early arithmetic competencies. Additional consideration of children’s knowledge of *zero* in the third step did not improve the regression model. Thus, results indicated that children’s understanding of the cardinality of small numbers might be associated with their language (vocabulary) skills. However, as soon as more domain-specific predictors were considered, the latter (i.e., counting skills and finger knowledge) seemed to overrule the influence of language.

As regards the relevant predictors, these findings are in line with earlier findings (e.g., Fuson, 1988; Gunderson et al., 2015) arguing for the importance of domain-specific abilities for the acquisition of the cardinality of small numbers. Moreover, our findings at least partly fit those of Negen and Sarnecka (2012), suggesting a more general influence of language skills on children’s understanding of cardinality (as reflected by the significant zero-order correlation of vocabulary and both children’s *zero* knowledge as well their understanding of cardinality). However, in the present study, visuospatial abilities were not a relevant predictor for children’s understanding of the cardinality of small numbers. This seems to be in contrast to the findings of Pixner et al. (2017) who observed an association between visuospatial abilities and basic numerical competencies in kindergartners. Yet, a closer look at the study reveals at least two possible reasons for these differences. First, in the present study we specifically focused on investigating children’s understanding of the cardinality of small numbers, whereas Pixner et al. (2017) measured a broader concept of basic numerical abilities. Second, these differential results may be related to the age of the present sample. Children in our study were on average 1.6 years younger than those assessed in the study of Pixner et al. (2017). Therefore, one might speculate that visuospatial abilities only gain significance for numerical development at a later point in time. There is tentative evidence corroborating this hypothesis. On the one hand, the children examined in studies suggesting the influences of visuospatial abilities on numerical development (as described in more detail in the introduction, e.g., Siegler and Booth, 2004; Gunderson et al., 2012) were again older than the children of the present sample. Additionally, a recent review indicated that the associations of visuospatial and numerical representations become more pronounced with increasing age (McCrink and Opfer, 2014; Newcombe et al., 2015, for a review on the intertwined development of spatial and numerical competencies). In sum, this asks for future longitudinal studies evaluating specifically the interrelations and differential influences between visuospatial abilities and basic numerical competencies in children’s cognitive development.

Importantly, the present study only represents a first step toward a better understanding of children’s mastery of the concept of *zero*. Future studies are needed to further increase our knowledge on the acquisition of this important concept. An avenue for such studies may be to consider indefinite numeric quantifiers such as *none* and *nothing* and to evaluate their role in the acquisition of the concept of *zero*. Children in kindergarten may more likely be faced with the words *none* and *nothing* than with *zero* and need to integrate and combine these constructs with their concept of *zero*. In this context, it would be desirable to conduct multiple assessments of the understanding of *zero* but also of the cardinality of the numbers 1–7 to increase the reliability of the measures. Additionally, visuospatial abilities may not be considered a unitary construct but seen to involve several subskills and processes (e.g., Ansari et al., 2003). As such, it is certainly premature to suggest that visuospatial abilities are unrelated to numerical abilities (cf. Newcombe et al., 2015). Instead, it would be interesting to assess the different aspects of visuospatial abilities in future studies to better understand which aspects are and which are not related to numerical abilities, in more detail.

Finally, it is important to not overstate the observed non-significant influences of vocabulary and visuospatial abilities in our final regression models as suggesting that these variables would not be important for children’s numerical development in general and their understanding of the concept of *zero* as well as the cardinality of small numbers in particular. There is considerable evidence for the critical influence of these variables (e.g., Ansari et al., 2003; Barner et al., 2009; Negen and Sarnecka, 2012) and we observed a significant influence of vocabulary on both children’s understanding of the concept of *zero* as well as the cardinality of small numbers before considering domain-specific numerical predictors in the regression models. As such, it seems a question of proximity between the predictor and the criterion variable that needs to be considered.

In other words, when controlling a predictor (e.g., vocabulary) for a more proximal variable (e.g., counting abilities) makes the predictor non-significant, that does not necessarily mean it is not an important causal predictor of the criterion variable (in this case *zero* knowledge and understanding of the cardinality of small numbers). Even though vocabulary and visuospatial abilities may not be considered as immediate proximal causes of the learning of number words or the acquisition of the concept of *zero*, the influences of these cross-domain variables exist at different levels of how we conceptualize the learning process. Counting ability, for instance, may be considered a direct prerequisite for acquiring cardinality knowledge. This sets it as a more proximal and direct cause, which needs to be dealt with in a somewhat separate way from the broader aspects of cognitive development like general vocabulary and visuospatial abilities.

Therefore, our argumentation is not about downplaying the influences of less proximal cross-domain cognitive abilities on children’s numerical development. However, evaluating the influences of broader cross-domain and proximal domain-specific variables as well as their potential interplay would require a longitudinal dataset for which direct versus indirect effects of the respective predictors as well as potential mediating effects can be evaluated. Therefore, future longitudinal studies would be desirable that not only consider more specific aspects of cross-domain abilities but also allow the evaluation of the direct as well as indirect influences of proximal domain-specific numerical and broader cross-domain variables as well as their interplay.

## Conclusion and Perspectives

The present study aimed at evaluating the differential influences of cross-domain abilities (i.e., language and visuospatial skills) and domain-specific basic numerical abilities (i.e., counting, number identification, and finger-based representations) on kindergartners’ understanding of the concept of *zero* and the cardinality of small numbers. In sum, our results indicated that children’s understanding of both the concept of *zero* and the cardinality of small numbers was associated significantly with their language skills. However, this association became insignificant as soon as domain-specific numerical predictors were considered. This substantiates the relevance of basic numerical competencies for children’s early numerical development. However, as discussed above, the present study could not identify whether the relevance of cross-domain and domain-specific variables for children’s numerical development differs over time. It might be that in some periods (for instance, during early numerical development in kindergarten), domain-specific numerical competencies are specifically important when children need to build up an abstract knowledge of number magnitudes. As indicated by the use of fingers for counting and initial arithmetic, building up this knowledge may be bound more closely to domain-specific aspects. Later on, when children will have successfully understood the cardinality of number magnitudes, cross-domain abilities may gain influence (e.g., Geary et al., 2017). As such, future longitudinal studies on children’s early numerical development in kindergartens would be desirable to evaluate these claims.

## Ethics Statement

RCSEQ, Research Committee for Scientific and Ethical Questions from the UMIT, Hall in Tyrol. This study was carried out in accordance with the recommendations of ‘RCSEQ, Research Committee for Scientific and Ethical Questions from the UMIT, Hall in Tyrol’ with written informed consent from all subjects. All subjects gave written informed consent in accordance with the Declaration of Helsinki. The protocol was approved by the ‘RCSEQ, Research Committee for Scientific and Ethical Questions from the UMIT, Hall in Tyrol.’

## Author Contributions

SP substantial contributed to the conception or design of the work and the acquisition, analysis, or interpretation of data for the work. VD drafted a part of the work. KM drafted a part of the work and revised it critically for important intellectual content.

## Conflict of Interest Statement

The authors declare that the research was conducted in the absence of any commercial or financial relationships that could be construed as a potential conflict of interest.
